# Chain Breaking Antioxidant Activity of Heavy (S, Se, Te) Chalcogens Substituted Polyphenols

**DOI:** 10.3390/antiox8100487

**Published:** 2019-10-16

**Authors:** Caterina Viglianisi, Stefano Menichetti

**Affiliations:** Department of Chemistry “Ugo Schiff”, University of Florence, Via Della Lastruccia 3-13, 50019 Sesto Fiorentino, Italy; caterina.viglianisi@unifi.it

**Keywords:** sulfur, selenium, tellurium, polyphenols, chain breaking antioxidants

## Abstract

Polyphenols are probably the most important family of natural and synthetic chain-breaking antioxidants. Since long ago, chemists have studied how structural (bioinspired) modifications can improve the antioxidant activity of these compounds in terms of reaction rate with radical reactive oxygen species (ROS), catalytic character, multi-defence action, hydrophilicity/lipophilicity, biodistribution etc. In this framework, we will discuss the effect played on the overall antioxidant profile by the insertion of heavy chalcogens (S, Se and Te) in the phenolic skeleton.

## 1. Introduction

**Figure 1:**
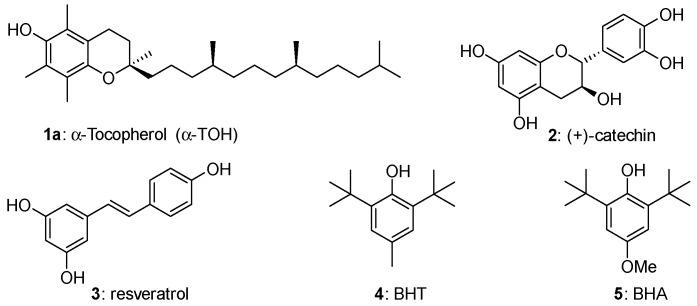
Structure of model natural and synthetic phenolic antioxidants. BHT—butylated hydroxytoluene; HBA—butylated hydroxyanisole.

Phenols represent the more important family of chain-breaking antioxidants able to inhibit or retard the autoxidation of organic compounds including biomolecules in living organisms and handcraft materials [1,2,3,4,5]. Thus, α-tocopherol [6,7,8,9,10,11] (α-TOH, 1a), the more potent lipophilic antioxidant known in nature (and the more abundant component of Vitamin E), catechin (2), a potent antioxidant member of the flavonoids family [12,13,14,15] (often indicated as Vitamin P) and resveratrol [16,17,18] (3) can be paradigmatically indicated as models for the thousands of natural polyphenols involved in the protection of living tissues from oxidative stress (Figure 1). Indeed, the dangerous increasing of oxidants species concentration in tissues, such as Reactive Oxygen Species (ROS), has been associated with many different pathologies and aging itself [1,2,3,4,5]. On the other hand, butylated hydroxytoluene (BHT, 4) or butylated hydroxyanisole (BHA, 5) represent the core skeleton of phenolic antioxidant additives worldwide used for increasing shelf life and protecting from autoxidation plastics, lubricants, oils, solvents and many other handwork materials [19,20,21] (Figure 1).

Although the situation at the biological level is, obviously, much more complex, the more important and peculiar feature of phenolic antioxidants is their ability in transferring a hydrogen atom (H•) from the ArOH to the radical chain propagating species, typically a peroxyl radical ROO•, thus, blocking or, at least, retarding the oxygen mediated autoxidation of organic molecules. Equations (1)–(4) report a simplified scheme of autoxidation that is applied when R is one of the unsaturated chains of a cell membrane, as well as when R is the aliphatic chain of a polyolefin etc. [22,23].

R ⟶ R•(1)

R• + O_2_ ⟶ ROO• (2)

ROO• + R-H ⟶ ROOH + R•(3)

ROO• + ArOH ⟶ ROOH + ArO•(4)

The formation of the first radical (R•, Equation (1)) can be the result of the natural metabolism of a living organism, or due to an oxidative event associated with heat, radiation, injury, inflammation etc. If the radical is formed in the presence of molecular oxygen, a very fast reaction occurs (the kinetic constant for this reaction is similar to that of diffusion ≥ 10^9^ M^−1^s^−1^) with the unavoidable formation of a peroxyl radical ROO• (Equation (2)). Peroxyl radicals are reactive enough to extract a H• from organic molecules (for example from allylic positions of unsaturated fatty acids) causing the formation of a hydroperoxide ROOH and a new radical R• (Equation (3)) in the propagation step of the autoxidation process.

Substituted phenols ArOH can react with peroxyl radicals faster than the great part of organic molecules R avoiding its oxidation. This reaction affords hydroperoxides ROOH and aryloxy radicals ArO• (Equation (4)). The latter, if properly substituted, are stable enough to not extract a H• from the surrounding. Thus, the chain oxidation is broken, or at least retarded, until the phenol can continue in its action of sacrificial reductant. Typically, antioxidant phenols are able to quench two ROO• radicals (i.e., the number *n* of ROO• quenched is 2). This can occur by the reaction of ArO• with a second equivalent of ROO•, as in the case of tocopherols, or with the extraction of a second H•, as in the case of catechin and related polyphenols. In both cases, after the second reaction, the phenol is transformed in a non-radical species, unable to further propagate the autoxidation or other undesired oxidative paths. Hydroperoxides, ROOH are unavoidably formed during the process (Equations (3) and (4)) and represent, in turn, potentially dangerous oxidants. These species require quenching mechanisms not involving phenolic species, and, as we will describe in the next chapters, heavy chalcogens derivatives containing, mainly Se and Te, can efficiently serve for this scope.

The efficiency of phenols as chain-breaking antioxidants depends upon the Bond Dissociation Enthalpy (BDE) of ArO-H bond involved in the H• transfer process and upon the kinetic constant (*k_inh_*) of the reaction of ArOH with ROO• (Equation (4)) [22,23]. Indeed, for homologous families of phenols, BDE and log *k_inh_* have a linear correlation: the lower the BDE, the higher the *k_inh_*, the faster the H• transfer process, the better the chain antioxidant activity. Thanks to the pioneering work of K.U Ingold, G. F. Pedulli and co-workers, solid procedures for measuring BDE, by Electron Paramagnetic Resonance (EPR) techniques, and kinetic constants *k_inh_*, quantifying the O_2_ consumption in the autoxidation of model hydrocarbons, of differently substituted phenols have been settled out. This allowed elucidating the role of phenolic structure and reaction media on the antioxidant activity and, to design more potent derivatives [22,23].

**Figure 2:**
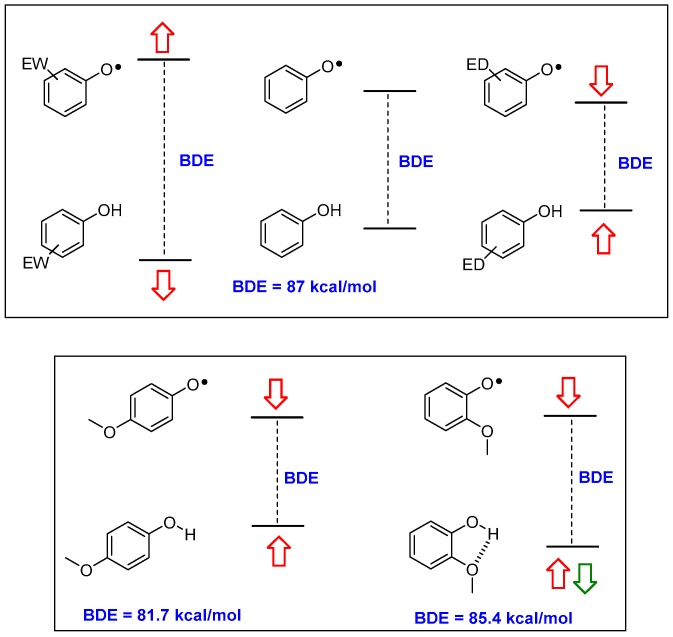
Electronic (red arrows) and intra-HB (green arrows) effects in modifying the ArO-H BDE value in substituted phenols. ED—electron-donating; EW—electron-withdrawing; BDE—Bond Dissociation Enthalpy.

Briefly, parent phenol PhOH has a BDE of 87 kcal/mol; a value too high for ensuring a fast reaction with ROO• (BDE of ROO-H is 88 kcal/mol). Additionally, once formed, parent phenoxyl radical PhO• could extract a H• from organic molecules (for example bis-allylic carbons in poly-unsaturated fatty acids have a C-H BDE of 89 kcal/mol) acting as the actual promoter of the chain oxidation. Considering the modifications required for lowering BDE (i.e., increasing the *k_inh_*) it must be considered that while a phenolic OH residue is, overall, an electron-donating (ED) group, the corresponding phenoxyl radical O•^−^ group, is, instead, strongly electron-withdrawing (EW). Hence, substituents with an EW character are able to stabilize the phenol ArOH, while ED groups are able to stabilize the ArO• (and vice-versa). Thus, ED groups destabilize the ArOH level and stabilize the ArO• level with an overall decreasing of the BDE, as expected in a good chain-breaking antioxidant (Figure 2). Of similar importance is the role played by the stabilization of ArOH by the formation of inter- or, above all, intramolecular hydrogen bond (inter-HB and intra-HB). Thus, an ED group able to act also as an intra-HB acceptor (for example, a methoxy group) will decrease the BDE more efficiently when in *para* than when in *ortho* position, since in the former situation, the ‘electronically driven’ destabilization of ArOH plus stabilization of ArO•, is reduced by the intra-HB stabilization of the ArOH (Figure 2).

**Figure 3:**
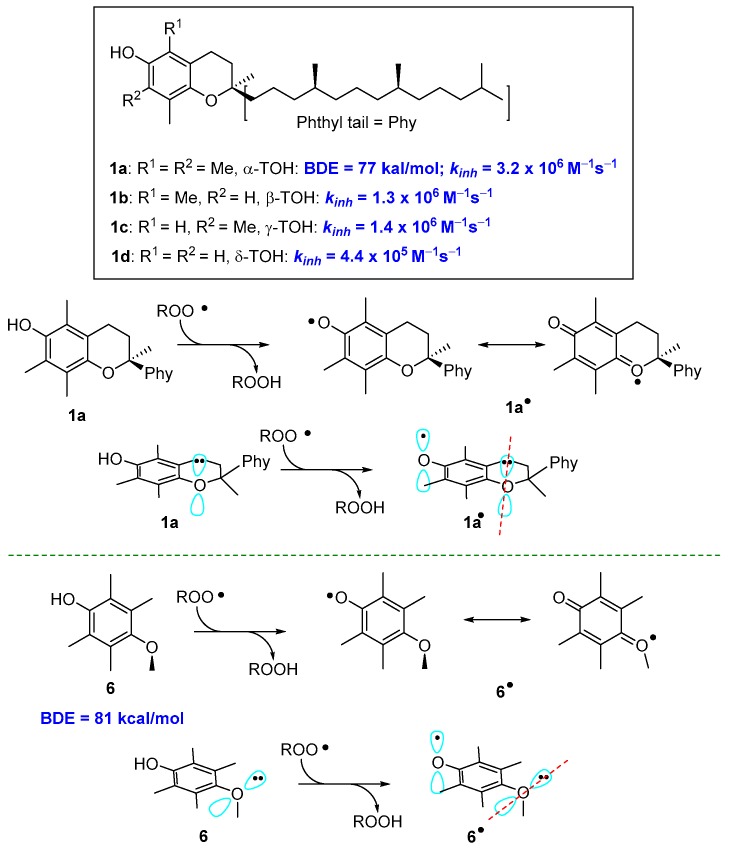
Role of conformations on resonance stabilization of ArO•.

These general concepts help to rationalise why α-TOH (1a, Figure 1) is the more potent natural lipophilic chain-breaking antioxidant. The aromatic phenolic ring has four ED alkyl groups and an alkoxyl oxygen *para* to the phenolic OH. Calculated BDE for α-TOH is 77 kcal/mol with a *k_inh_* of 3.2 × 10^6^ M^−1^s^−1^. Indeed β-, γ, and δ-TOH (1b–c, Figure 1) with two or one methyl groups are, in turn potent polyphenolic antioxidants, yet showed a lower *k_inh_* than fully methylated 1a (Figure 3). Additionally, in tocopherols, alkoxy oxygen is part of a six-membered benzo-fused chromane ring. This situation forces the oxygen lone pair to lay almost parallel to the aromatic π system, maximizing the ability of resonance stabilization of α-TO• radical. To better understand how conformations can modify the ArO• stability, the 2,3,5,6-tetramethyl-4-methoxy phenol 6, bearing for alkyl and a para-alkoxy group, has a BDE higher than those of 1a–d. In fact, the steric hindrance brought about methyl groups on C5 and C6 favours the conformation with the Oxygen-Methyl bond laying almost perpendicular to the aromatic ring. In this situation, the lone pair on methoxy oxygen in no longer in the right position for an efficient ArO• stabilization (Figure 3).

**Figure 4:**
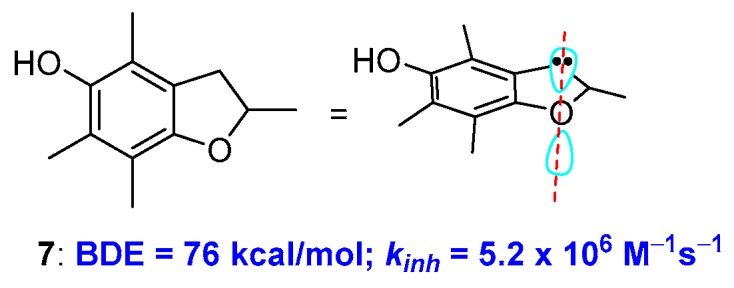
Effect of ring size on BDE of benzo-fused oxygen heterocycles.

To further demonstrate how conformations can contribute to the rate of H• transfer process, Ingold reported that the insertion of the *para*-alkoxy oxygen in a five-membered ring, i.e., moving from a chromane to a dihydrobenzo[b]furane system, causes a further increase of *k_inh_*. This was rationalised considering that a five-membered ring is more rigid and, consequently, increasing the alignment between the oxygen lone pair and the aromatic π system. This observation was used for the preparation of very potent chain breaking antioxidants. For example, compound 7 showed a BDE of 76 kcal/mol of and a *k_inh_* of 5.0 × 10^6^ M^−1^s^−1^ discernibly better than the α-TOH performances [24,25,26] (Figure 4). Noteworthy, similar conformational considerations have been exploited for chalcogens substituted phenols [27,28] [8P, 8P′].

**Figure 5:**
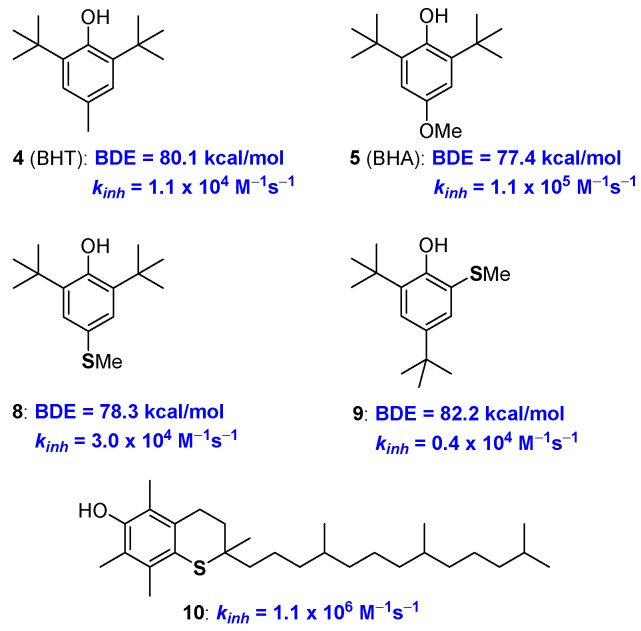
Performances of Sulfur analogues of BHA 8, 9 and all*rac*-1-thio-α-tocopherol 10.

Following these general criteria and considering the simplicity and cost/efficiency issues, synthetic BHT (4) and BHA (5) were selected as the core skeletons of the worldwide-used antioxidant additives in plastics, lubricants, oil etc [19,20,21]. In fact, the aromatic rings are substituted with ED groups and in BHA a methoxy group is placed *para* position to the phenolic OH. This allows a quite low BDE (BDE for BHT and BHA are 77 and 80 kcal/mol respectively, see Figure 5) and a fast reaction with ROO• as required for the protection of materials from auto-oxidation. BHT and BHA are in turn able of quenching two ROO• equivalents (*n* = 2) and, additionally, the stability of the corresponding ArO• phenoxyl radicals is further increased by the steric protection brought about by the two *ortho* tert-butyl groups.

It must be underlined that the BDE lowering of the ArO-H bond, obtainable with the introduction of the right substituent(s) in the right position(s), cannot push as low as possible. In fact, with BDE values lower than 72 kcal/mol ArO-H could directly react with molecular oxygen to give a radical cation ArO-H•^+^ and superoxide radical anion O_2_•^−^. Such a phenol would be unstable to air and useless as an antioxidant. Thus, the design of new antioxidants requires the ability to prepare compounds able to react very fast with ROO• and other dangerous ROS, giving stable safe products but unable to react with O_2_.

The fine-tuning of phenols structure/antioxidant activity relationship has been investigated in detail by many groups around the world with several excellent results in term of performances, stability and access feasibility [29,30,31,32,33,34]. In this focussed review, the main achievements reached using heavy chalcogens (S, Se and Te) as substituents of the phenolic skeleton will be considered and discussed. The aim of the review is to report how the stereoelectronic features of chalcogen substituents can modify the chain-breaking antioxidant ability of phenols, and how the introduction of chalcogens can be exploited for the preparation of new potent antioxidants and for a better understanding of the red-ox biological processes where chalcogen containing phenols are involved with. Reaction media (solvent, pH, additives, etc.) can dramatically modify the antioxidant performances of phenols [35,36,37,38,39]. However, insights acquired reaction media effects remain completely true for chalcogens substituted phenols, thus the discussion about the role of S, Se or Te on the antioxidant activity can be carried out independently. Additionally, several different procedures to measure and quantify the antioxidant activity of phenols are available nowadays [40,41,42]. In this review, we will consider exclusively the chain-breaking activity of phenols evaluated using the rigorous physicochemical approaches settled out by K. U. Ingold, G.F Pedulli [22] and L. Engman (*vide infra*).

Research on chalcogens containing phenolic antioxidants has been developed in the last four decades or so, with new achievements on derivatives bearing, usually, one of the three elements. However, compounds with a similar structure, yet substituted with S or Se or Te, have been reported as well and studied for comparing the effect of each chalcogen. In the hope of facilitating the discussion and valorising similarities and differences, we have decided to describe initially the chemistry of sulfur-containing antioxidants, to then move to those having Se or Te.

## 2. Discussion

### 2.1. Sulfur Containing Phenolic Antioxidants

A solid quantification of the effect of sulfur substituents on BDE, and *k_inh_*, of phenolic antioxidants, is relatively recent [43]. Pedulli, Menichetti and co-workers reported that in compound 8 the thio-analogue of BHA, a *para*-thiomethyl group decreases the BDE of about 3.6 kcal/mol, a value that is lower than the 4.4 kcal/mol measured for a *para*-methoxy group. On the other hand, the *ortho*-thiomethyl group in 9 decreases the BDE of about 0.8 Kcal/mole being just 0.2 kcal/mol the contribution of an *ortho*-methoxy group (Figure 5). In other words, when in *para* position, the stabilization of ArO• and destabilization of ArOH, i.e., the overall ED character effect, is less important for a -SCH_3_ than for a -OCH_3_ group. Considering the *ortho*- substitution, the weaker intra-HB stabilization offered by an adjacent SCH_3_ group causes an overall superior decrease of BDE (0.8 vs 0.2 kcal/mol) moving from sulfur to oxygen [43].

However, taking into consideration that, for example, an *ortho*-methyl group decreases the ArO-H BDE of about 2.0 kcal/mol, the introduction of thioalkyl residues seems, neither in *para*- nor in *ortho*-position, convenient for increasing the chain breaking antioxidant activity of phenols. Indeed, similar consideration emerged after the synthetically demanding preparation of all-*rac*-1-thio-α-tocopherol 10, and related species, carried out by Ingold more than 30 years ago [44,45]. The substitution, in the benzo-fused chromane ring, of oxygen with sulfur (i.e., from 1a to 10) decreased the *k_inh_* to 1.1 × 10^6^ M^−1^s^−1^, which is about one third of that of 1a, demonstrating the reduced ability of the thiochromane *para*-sulfur atom in stabilizing tocopheryl radical (α-TO•) as quantified two decades after [43] (Figure 5).

**Figure 6:**
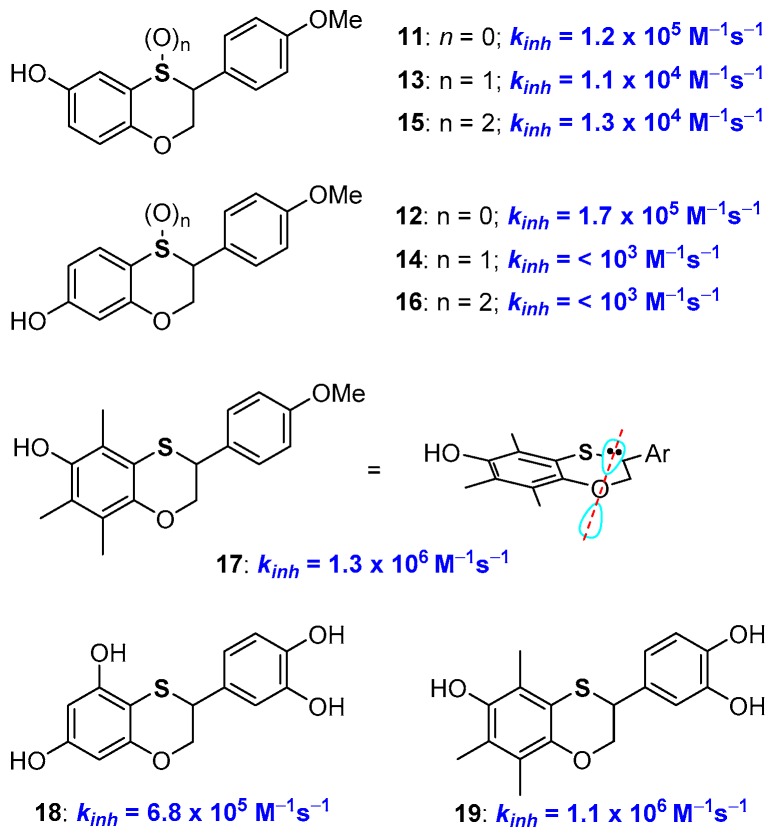
A selection of 2,3-dihydrobenzo[1,4]oxathiine multi-defence antioxidants.

We have reported a practical preparation of phenolic compounds with a 2,3-dihydrobenzo[1,4]oxathiine skeleton with valuable antioxidant activity [46,47,48,49,50]. The synthetic opportunity was exploited to prepare, for example, derivatives 11 and 12 with identical structures but a sulfur or an oxygen atom *para*- to the phenolic OH involved in H• transfer process (Figure 6). Indeed, *k_inh_* of 11 was higher than that of 12 boosting the better ability of a *para*-conjugated oxygen atom in stabilizing phenoxyl radicals ArO•.

Having the possibility to prepare a great variety of benzoxathiine antioxidants and to manipulate their structure allowed us to achieve a number of additional insights about the role of the sulfur atom on the activity of these polyphenolic compounds. For example, oxidation at sulfur [43,48], with the formation of either sulfoxides, like 13 and 14, or sulfones, 15 and 16, sensibly depletes the H• transfer ability of these species above all when the sulfur atom is directly conjugated with the phenolic OH. This was expected due to the transformation of the ED group (the sulfide) into an EW group (the sulfoxide or sulfone) that will be particularly effective when the sulfur atom is directly conjugated with the -OH/-O• group (i.e., like in compounds 14 and 16). Additional considerations emerged considering derivative 17, and related species, that possesses exactly the aromatic skeleton of α-TOH (1a, Figure 1) with a sulfur atom in the place of a CH_2_ in position 4. This compound showed a *k_inh_* of 1.3 × 10^6^ M^−1^s^−1^ and a BDE of 79 kcal/mol [51]. Thus, the introduction of the sulfur atom in position 4 decreases the chain breaking antioxidant activity as when it was inserted in position 1 (see derivative 10 Figure 5). This was rationalised considering that introducing a sulfur atom in a benzo-fused heterocyclic ring means increasing the flexibility of the system (due to the two long sulfur-carbon bonds). As demonstrated by an X-ray, at least in the solid-state, the benzoxathiine skeleton of 17 showed a reduced planarity when compared with chromane system of tocopherols. Thus, in 17, the endocyclic oxygen lone pair is less parallel to the aromatic π system than in 1a, causing a reduced ability in stabilizing the ArO• radical. In other words, introducing a sulfur atom in the chromane ring (i.e., having a benzoxathiine ring) means increasing the flexibility of the system i.e., in the opposite direction of increasing the antioxidant activity (compare Figure 4 and Figure 6, i.e., 1a vs 7 vs 17).

Benzoxathiines derivatives, like 11, 12 and 17, were prepared via a Diels-Alder reaction of a dienic *ortho*-thioquinone with electron-rich styrene used as a dienophile. Thus, the proper decoration of both reaction partners allowed the synthesis of derivatives possessing a tocopherol-like and a catechin-like structure [46,47,48,49,50,51]. Indeed, derivatives like 18 and 19 bring together the characteristics of two of the more important families of natural polyphenolic antioxidants (Figure 6).

Actually, the multi-defence antioxidant activity of hybrid derivatives is particularly appealing since the synergism between structurally different antioxidants is a mandatory requisite, at a biological level, for transforming high oxidant radical species into safe unreactive derivatives through a cascade of red-ox quenching processes [52,53,54,55,56,57,58,59].

**Figure 7:**
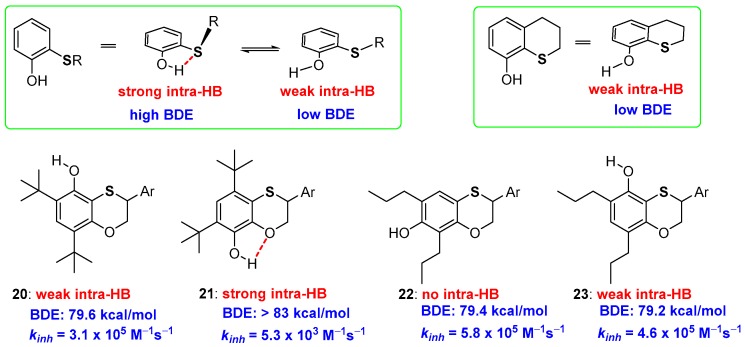
Conformational and substitution considerations on intra-HB strength of acyclic and cyclic *ortho*-thioalkyl substituted phenols, and benzoxathiines 20–23 prepared to quantify the role of this phenomenon on *k_inh_* and ArO-H BDE.

When studying the reactivity of benzoxathiine derivatives, we faced the problem to quantify the H• transferability of OH groups laying in *ortho*- or in *para*-position to the endocyclic benzoxathiine sulfur [60]. As defined, a thioalkyl residue reduces the BDE of an *ortho*-OH group of about 0.8 kcal/mol as the balance between ArOH/ArO• stabilization by ED effect and ArOH stabilization by intra-HB [43]. However, as it was reported long ago, a strong intra-HB with *ortho*-thioalkyl groups is possible only when the S-alkyl bond lays perpendicular to the aromatic phenolic plane [61,62,63,64,65]. When the S-alkyl bond is structurally forced (near) parallel to the phenolic ring the intra-HB strength sensibly decreases, hence it decreases the stabilization of ArOH and the ArO-H BDE (Figure 7). This phenomenon is typical of heavy chalcogens, while oxygen is able to give strong intra-HB almost independently upon the conformation considered. As we will discuss later on, this is directly related to “σ-hole and chalcogen-bond” issues that must be considered since to give a complete frame of S, Se and Te stereoelectronic contribution to H• transfer processes [61,62,63,64,65]. Measuring *k_inh_* and BDE of a set of different substituted benzoxathiines (20–23, Figure 7) with the OH group *ortho* or *para* to the sulfur or the oxygen atom of benzo-fused heterocycle, we could quantify this contribution, demonstrating that a sulfur atom inserted in a six-membered ring decrease the BDE of an *ortho*-OH group of about 3.1 kcal/mol [60]. Indeed, a very week intra-HB is observed, as validated by Infra Red (IR) spectra and *ab-initio* calculations, in compounds 20 and 23, this sensibly decreases the ArOH stabilization and also the corresponding BDE. As a matter of fact, boosting the evidence of a very week intra-HB, X-ray analysis of derivative 23, showed that in the solid-state, the phenolic proton lays far from the sulfur atom pointing in the direction of the *ortho*-alkyl group.

This observation was used to suggest a rationale for the cysteine-tyrosine post-transactional aryl-S bond formation in the galactose oxidase active site. This enzyme catalyses the oxidation with molecular oxygen, of the primary alcohol residue of galactose to the corresponding aldehyde and hydrogen peroxide. The catalytic cycle foresees the formation, on the tyrosine residue, of an *ortho*-thiosubstituted phenoxyl radical that represents the key oxidative intermediate of the whole process. We postulated that the rotation around the aryl-sulfur bond, modifying the BDE of the tyrosine OH (with the thioalkyl group perpendicular to the aromatic ring: strong intra-HB, stable ArOH high BDE; with the thioalkyl group on the plane of the aromatic ring: weak intra-HB, stable ArO• low BDE) facilitates the red/ox cycles. Probably, the arrival in the active site of the reduced galactose and its departure as oxidised galactose triggers the rotation around the aryl-sulfur bond modifying the red/ox potentials as required for the enzyme action [60].

**Figure 8:**
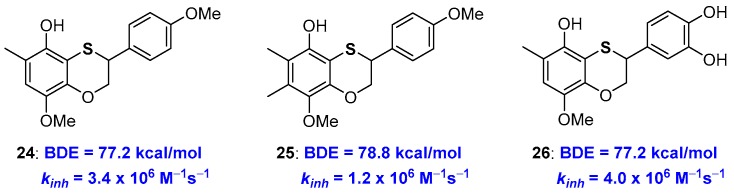
Optimized benzoxathiines phenolic chain breaking antioxidants 24–26.

At the same time, this observation indicates that in a sulfur atom inserted in a benzoxathiine ring, an efficient substituent to be placed *ortho*- to a phenolic OH involved in the H• transfer event. Indeed, exploiting this opportunity, we designed properly substituted derivatives and prepared very potent chain-breaking benzoxathiine antioxidants, for example compounds 24–26, with abilities near or superior to those of α-TOH 1a, also maintaining the catechin-like character [66] (Figure 8).

As already reported in the introduction [22,23], increasing the number of alkyl groups does not automatically mean increasing *k_inh_* (or decreasing BDE). In fact, chain breaking antioxidant 25 showed performances worse than 24 despite the additional methyl group on C7. In fact, this methyl laying *ortho* to the methoxy group disfavours, by steric hindrance, the conformation with the O-Me bond parallel to the aromatic ring required for an efficient ArO• stabilization (see Figure 3). Such steric repulsion is important enough to prefer a situation without substituents in position 7 that is the skeleton chosen for derivative 24 and catechin/tocopherol hybrid 26 both showing *k_inh_* higher than those of α-TOH 1a. As expected, compound 26 was capable of quenching four equivalents of ROO• radicals (*n* = 4) possessing both the tocopherol-like and the catechol-like portion operative [66].

**Figure 9:**
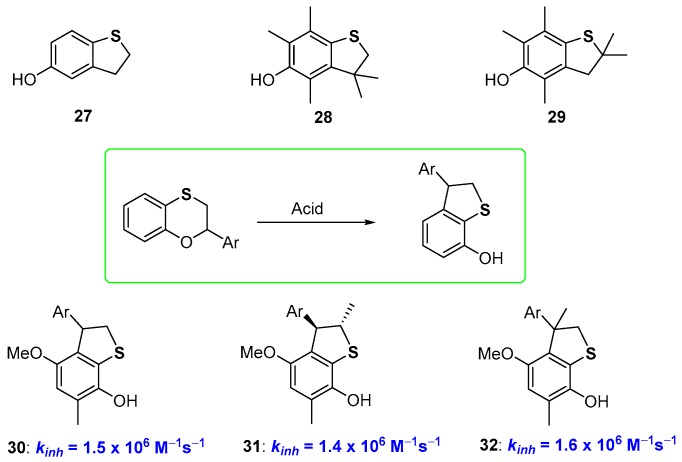
Hydroxy-2,3-dyhydrobenzo[*b*]thiophene antioxidants.

It has already been reported that moving from six to five-membered rings in phenolic species bearing benzofused oxygen substituted heterocycles means increasing the rigidity of the system, with a benefit on the chain-breaking antioxidant activity [24,25,28]. Actually, a similar trend was not observed for 5-hydroxydihydrobenzo[*b*]thiophenes of type 27–29 (Figure 9) which demonstrated to be potent chain-breaking antioxidants but did not overwhelm the performances of α-TOH. We could rationalise this result having developed a new procedure for the preparation of 7-hydroxydihydrobenzo[*b*]thiophenes based on an acid promoted transposition of benzoxathiines. Thus, these latter heterocycles, are, at the same time, an interesting skeleton for antioxidant polyphenols and starting material for new phenolic derivatives [67]. When carried out on structurally properly designed starting materials, the 2,3-dihydrobenzo[1,4]oxathiine → hydroxydihydrobenzo[*b*]thiophene transposition allows the preparation of potent chain breaking antioxidants like 30–32, taking advantage of the *ortho*-endocyclic sulfur effects discussed before, namely (i) the ED effect on ArO• stabilization by the *para*-alkoxy and *ortho*-sulfide groups; (ii) the weak ArOH stabilization by negligible intra-HB [67] (Figure 9).

As a matter of fact, moving from derivatives 24–26 to 30–32, i.e., decreasing the ring size of the benzofused heterocyclic ring, we observe a tiny decrease of *k_inh_* (compare data of Figure 8 and Figure 9). This was explained considering that in dihydrothiopene derivatives (like 27–32) the two long sulfur bonds allowed enough flexibility to vanish the expected increase in rigidity after ring constraint. Indeed, X-ray analysis of derivative 30 indicates that the dihedral angle among the aromatic and the heterocyclic ring is very similar to that measured in benzoxathiines like 23 or 24, supporting, at least in the solid-state, the above justification. However, it must be underlined that the transformation reported in Figure 9 used for the synthesis of compounds 30–32, represents the one-step conversion of a non-phenolic derivative with no antioxidant activity into a potent phenolic chain breaking antioxidant [67].

**Figure 10:**
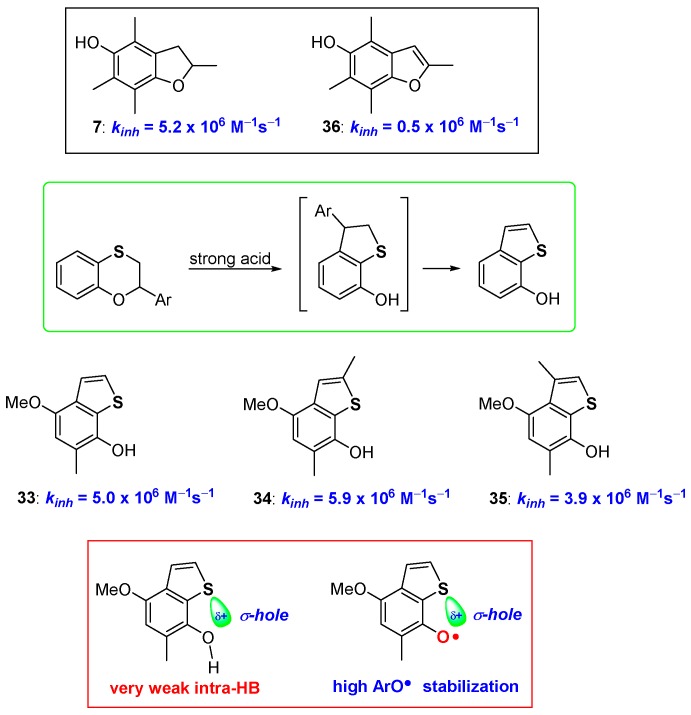
Effect of aromatization on chain breaking antioxidant activity of dihydrobenzo[*b*]furane/benzo[*b*]furane *vs* dihydrobenzo[*b*]thiophenes/benzo[b]thiophenes couples.

Even more interestingly, when a proper structural arrangement is respected and placed under harsher reaction conditions, a second rearrangement could take place transforming 7-hydroxydihydrobenzo[*b*]thiophenes, like 30–32, into the corresponding aromatic 7-hydroxybenzo[*b*]thiophenes 33–35 [68] (Figure 10). Many years ago, Ingold demonstrated that comparing the antioxidant activity of 5-hydroxydihydrobenzo[*b*]furanes (like 7, Figure 4) with the corresponding aromatic 5-hydroxybenzo[*b*]furanes (like 36, Figure 10) an important decrease of the *k_inh_*, almost an order of magnitude, can be observed [45]. This was explained by considering that, with aromatization, the lone pair on oxygen, required for ArO• stabilization, is actually engaged in the 10 electrons aromatic system. Thus, despite the increased rigidity and perfect planarity reached, aromatization caused a significant decrease of the H• transfer process rate [45] (Figure 10).

Surprisingly, 7-hydroxybenzo[*b*]thiophenes 33–35 showed *k_inh_* higher than those of the corresponding 7-hydroxydihydrobenzo[*b*]thiophenes 30–32, indicating that when moving from oxygen to sulfur, aromatization becomes beneficial for the chain breaking activity [68]. To rationalise this difference, we can consider that aromatization brings the complete planarity of the system, hence a very weak intra-HB, as confirmed by IR measurements, is present in derivatives 33–35. However, this does not seem enough to justify the observed *k_inh_* increasing. On the other hand, it has been reported that sulfur σ-hole, i.e., the electron-poor zone pointing along the direction of the carbon-sulfur bond, becomes more important with aromatization moving from dihydrothiophenene to thiophene systems [69,70,71,72,73,74,75,76]. Actually, the σ-hole is responsible of the very weak intra-HB at ArOH level, but it could be also responsible for an extra stabilization of the vastly electron-rich area around the phenoxyl radical ArO•. This speculation was corroborated by *ab-initio* calculations that indicated a similar, yet numerically more significant effect, moving from sulfur to selenium and tellurium, exactly the trend expected when a chalcogen-bond effect is operative. Indeed, we have suggested that, all the peculiar observations done on the *ortho*-thiosubstituted phenolic compounds prepared so far (role of conformations, intra-HB strength, ArOH/ArO• stabilization) can be rationalised only taking in the proper consideration the role that chalcogens σ-hole plays in all these mentioned features [69,70,71,72,73,74,75,76] (Figure 10).

### 2.2. Selenium and Tellurium Containing Phenolic Antioxidants

**Figure 11:**
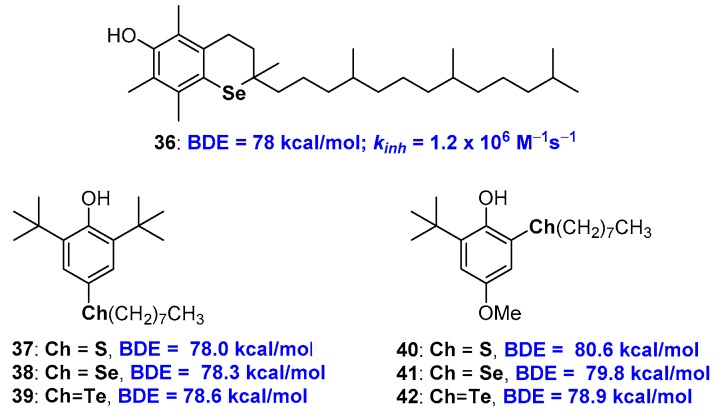
all*rac*-1-Seleno-α-tocopherol 36, and model chalcogen substituted polyphenols used to quantify the substitution contribution on chain breaking activity.

The chemistry of selenium and tellurium phenolic antioxidants has received a crucial contribution with the work of Professor L. Engman and co-workers. Actually, as a result of a collaboration between this group and the group of Professor G.F. Pedulli, the synthesis was published, very demanding indeed, and the evaluation of the antioxidant activity of all*rac*-1-seleno-α-tocopherol 36 [77] (Figure 11). Compound 36 showed a *k_inh_* of 1.2 × 10^6^ M^−1^s^−1^ with a BDE of 78 kcal/mol almost superimposable to those of the corresponding sulfur analogue 10, see Figure 5, being both less potent than natural α-TOH 1a. More recently, the same authors were able to quantify the contribution that acyclic alkyl chalcogen substituents, as for example in derivatives 37–42, have on BDE of phenolic antioxidants as function relative chalcogen/-OH relative *ortho* or *para* position [78,79] (Figure 11). Thus, when chalcogen alkyl substituents are in *para* position, the better performances, i.e., the higher decrease of BDE, are produced by sulfur substituents, then selenium and tellurium. This can be explained considering that ED the effect in ArO• stabilization decreases with increasing atomic weight. On the other hand, an opposite trend is observed for alkyl chalcogen substituents in *ortho* position. In this case, the contribution of the chalcogen σ-hole seems to be the key to rationalising the result. In fact, a weaker intra-HB and a better ArO• stabilization are expected when moving from sulfur to tellurium due to the increasing chalcogen-Bond effect with atomic weight increasing [78].

For Se and Te substituted phenolic antioxidants, we can consider the different stereoelectronic contributions listed for sulfur derivatives. However, such discussion would be limited without considering the peculiar characteristic of many Se and Te substituted phenolic antioxidants i.e., the opportunity to be regenerated by a sacrificial reductant, typically a thiol, thus allowing to be used in a catalytic amount.

**Figure 12:**
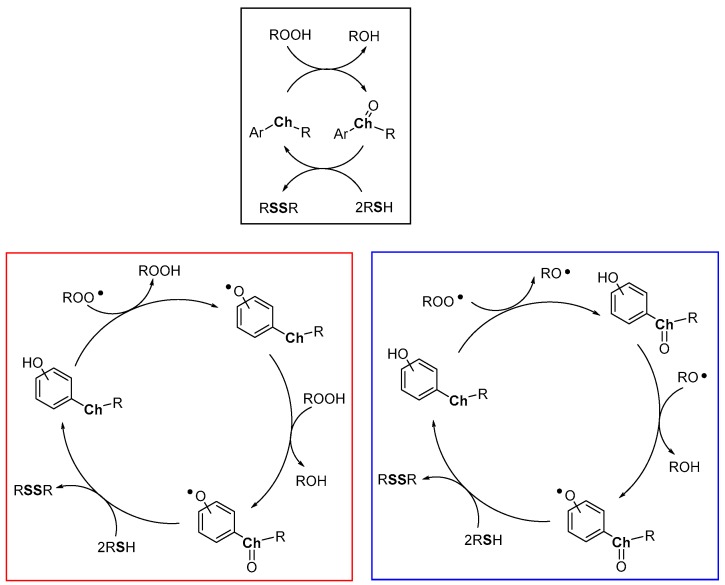
Heavy chalcogens (Se, Te) possible catalytic cycles (black frame, red frame and blue frame) in the presence of hydroperoxides or peroxyl radicals as oxidants and a sacrificial thiol as co-antioxidant.

Heavy chalcogens (Se, Te) are able to react with hydroperoxides to give a chalcogen oxide which, in turn, can be reduced back by two equivalents of a thiol with the formation of a disulfide and water (Figure 12, black frame). Due to the different red/ox potentials of chalcogens, this reaction is typically operative with tellurium derivatives, it can be operative with selenium derivatives, with rates significantly depending upon the whole structural features of the compound, while, generally, it occurs at a negligible rate with sulfur derivatives. With the obvious generalization, this is the process carried out by glutathione peroxidase (GPx) enzyme, and in particular by the selenocysteine residue operating in the enzyme active site. Indeed, GPx operates quenching hydroperoxides (ROOH) while a thiol (reduced glutathione, GSH) is oxidised to a disulfide [80,81,82,83,84,85] (oxidized glutathione, GSSG). However, studying the reaction of peroxyl radicals ROO• with heavy chalcogen substituted phenols, we must consider that two oxidizable sites are present and two different oxidative processes can take place: (i) the direct H• transfer from ArOH and, (ii) the oxidation at chalcogen (mainly Se and Te). Both the H• transferability and the oxidation at chalcogen will depend by the whole molecular skeleton including the mutual influence of these groups. Additionally, the H• transfer process from ArOH to ROO• generates a hydroperoxide ROOH, able, in turn, to oxidize the chalcogen atom.

Simplifying the possible situations, we can imagine that the ROO• initially extracts a H• from the phenolic OH, as in all the examples seen up to now, with a rate depending by the ArOH skeleton including the effect of the chalcogen substituent (Figure 12, red frame). Then, the ROOH formed can oxidize the chalcogen to the corresponding chalcogen oxide (Ch=O). Eventually, the thiol regenerates the starting phenol that behaves as a catalyst being the thiol the real consumed co-antioxidant.

However, in many cases, the substitution pattern of the ArOH cannot justify the observed rates of ROO• depletion, with *k_inh_* ≥ 10^7^ M^−1^s^−1^. Thus, recently, a different mechanism has been suggested. In this new scenario, the initial reaction of ROO• occurs at chalcogen with the formation of Ch=O and an alkoxyl radical RO• (Figure 12, blue frame). This latter, extracts, typically in the solvent cage, a H• from the phenolic OH giving the ArO• and alcohol (ROH). Eventually, the thiol reduces the chalcogen oxide and the phenoxyl radical regenerating the phenolic antioxidants. In this way, the H• transfer process can be much faster than those described in the red frame and much less sensitive to ArOH whole substitution pattern, in particular when the phenolic OH and the chalcogen substituent where the RO• is formed are adjacent.

It must be underlined that, independently upon the mechanism operative, heavy chalcogen containing antioxidants seems particularly indicated at the biological level being able of quenching peroxyl radicals (ROO•) and hydroperoxides (ROOH) giving water, alcohols and disulfides as the only reaction products. However, depletion of endogenous thiols can, in turn, be a potentially dangerous situation.

For many phenols containing heavy chalcogens, the antioxidant activity and, in particular, their ability to act as catalytic antioxidants has been measured quantifying, by HPLC, the amount of conjugated diene formed from a polyunsaturated fatty acid in the presence of a radical initiator and molecular oxygen [86]. Thus, the amount of conjugated diene formed in the presence of the substrate under study, until its consumption, gives the inhibited rate of peroxidation (*R*_inh_) and the inhibition time (*T*_inh_) that taken together represent a solid quantification of the phenol ability to block chain oxidations. For example, under the standard conditions used (chlorobenzene as solvent, linoleic acid > 30 mM, antioxidant 40 μM) α-TOH (1a) showed a *R*_inh_ ≈ 25 μM h^−1^, and *T*_inh_ ≈ 90 min [87,88]. If the measure is carried out in a two phases system in the presence of an excess of a proper thiol, usually N-acetyl cysteine (NAC) 1 mM, it is possible to verify the catalytic activity verifying the increase of the inhibition time *T_inh_*. For example, for α-TOH, *T_inh_* and *R_inh_* are insensitive to the presence of a co-antioxidant thiol. Indeed, it is well known that thiols are unable to regenerate tocopherols after oxidation. For tellurium containing phenols, carrying on the measure in the absence of a co-antioxidant thiol, means not only that we do not observe any increase of *T*_inh_, but quite low *R_inh_* is measured. In other words, it seems that, without the reducing thiol, it also vanishes the ability of the tellurium substituted phenol to react with ROO•. This phenomenon has been explained considering that commercially available linoleic acid contains variable amounts, increasing with storing, of the corresponding hydroperoxides formed by autoxidation. As depicted in Figure 12 black frame, hydroperoxides are able to oxidize the tellurium derivatives to the corresponding telluroxides. Thus, in the absence of a thiol, the measured *R_inh_* is not that of the Tellurium substituted ArOH, yet that, much worst, of the corresponding a telluroxide R_2_Te=O [28,79,87,88,89,90,91].

**Figure 13:**
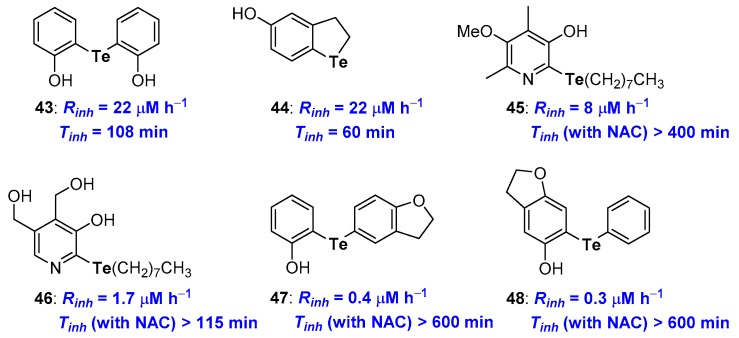
A selection of potent Tellurium containing phenolic antioxidants prepared so far.

Focussing on Tellurium substituents (Figure 13), since the first studies on symmetrical diarylhydroxy tellurides like 43 [86], it was clear how *para-* and, above all, *ortho*-hydroxy derivatives work quite well with *R*_inh_ and *T*_inh_ better than that of α-TOH. Inserting the Tellurium atom in a five-membered benzo-fused ring, like in 44, ensured a good *R*_inh_ while *T*_inh_ was quite short (22 μM h^−1^ and 60 min) [28]. More recently, phenolic skeletons that allow a very good chain breaking antioxidants, independently upon the presence of a chalcogen atom have been considered [78,79]. For example, compound 45, with several other structural related derivatives, or compound 46, bearing the Te atom on the skeleton of pyridoxine (Vitamin B6) have been prepared and their antioxidant activity studied [87,88,90,91] (Figure 13).

Results obtained with these phenolic derivatives follow the above-discussed trend, thus compound 45 showed *R*_inh_ better than α-TOH and a very good catalytic activity in the presence of NAC with *T*_inh_ ≥ 400 min [91]. On the other hand, tellurated Vitamin B6 analogue 46 showed an even better *R*_inh_ yet a worse catalytic activity [87]. For these compounds also, the presence of a co-antioxidant thiol is mandatory to maintain both activities. Recently, Prof Engman merging the above observations, designed and prepared a set of very potent diaryl telluro derivatives, like 47 and 48, [89] (Figure 13) whose structure allowed the synergetic action of the Te residue on the OH group H• transfer process aptitude, and the OH group in the oxidation potential of Tellurium. Thus, exploiting the ED donating effect of the oxygen atom in a five-membered benzo-fused ring and the whole system conjugation, it was possible to maximize the reaction of ROO• at Te followed by the H• transfer to RO• as described in the blue frame of Figure 12. As a matter of fact, measured *R*_inh_ (0.4 and 0.3 μM h^−1^) and *T*_inh_ (up to 600 min) for 47 and 48 were the best ever reported. It is worth nothing that the *R*_inh_ performance of these derivatives is only partially lost in the absence of co-reductant thiol. For example, without any co-antioxidant thiol, derivative 48 showed *R*_inh_ = 23 μM h^−1^ and *T*_inh_ = 136 min, performances superimposable to that of α-TOH confirming the success of the overall structural design [89].

**Figure 14:**
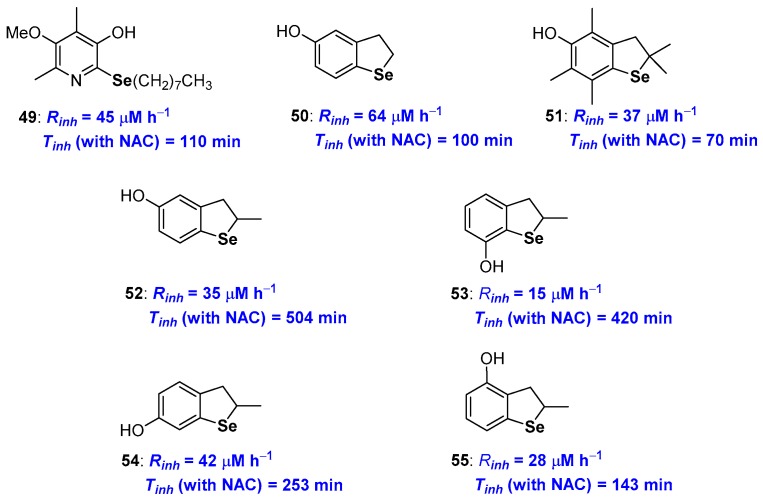
A selection of Selenium containing phenols prepared so far.

Selenium substituted phenols showed performances in between those of analogues containing sulfur or tellurium, as expected for the intermediate red/ox profile of Selenium. Indeed, selenotocopherol 36 (see Figure 11) was a chain breaking antioxidant very similar to the sulfur analogue 10 (see Figure 5) both showing performances worse than natural α-TOH (see Figure 1). A similar trend was observed for derivatives 36 and 41 [78,79] (Figure 11) as well as selenopyridinol 49 [90] (Figure 14) that showed a *R*_inh_ higher than those of the corresponding tellurium derivatives and very similar to the sulfur ones. Moreover, none of these compounds showed an efficient catalytic activity with inhibition times *T*_inh_ (and inhibition rates *R*_inh_) almost independent upon the presence of a co-antioxidant thiol. A great effort has been devoted to rationalizing the activity of dihydrobenzo[*b*]selenophenes of type 50–55 [28,92,93,94,95,96] (Figure 14). For all of these compounds it appears that the value of the inhibition rate *R*_inh_ can be rationalised considering the ED and *ortho*-OH effect (including the contribution of Se σ-hole in ArO• stabilization). Additionally, for all compounds, *R*_inh_ is almost insensitive to the presence of a thiol as co-reductant during the measure. Thus, for these selenium derivatives the oxidation to R_2_Se=O, due to the adventitious presence of hydroperoxides, is not able to modify the antioxidant kinetic profile either because it does not take place or because it occurs much slower than the reaction with peroxyl radicals. In a recent paper [94], Engman reported that 5-hydroxy- and 7-hydroxybenzo[*b*]selenophenes 52 and 53 (Figure 14), i.e., those with a *para*- and *ortho*- arrangement between the selenium atom and the OH group, are indeed able to be regenerated by a thiol with *T*_inh_ from four to five times longer (504 min and 420 min respectively) than those measured without the sacrificial thiol. For these two derivatives, the measured *R*_inh_ (15 and 20 μM h^−1^ respectively) are too fast to be justified considering an initial H• transfer process from the ArOH to the ROO• as in Figure 12 red frame. Also in this case, it was postulated that the first reaction is the oxidation at selenium by the peroxyl radical followed by the H• transfer ‘in the solvent cage’ as depicted in the blue frame of Figure 12. This allowed rationalising the order of reactivity, being derivative 53 (with chalcogen *ortho*-OH effects plus a site-favoured ‘in the solvent cage’ H• transfer) that showed the better performances (Figure 14).

**Figure 15:**
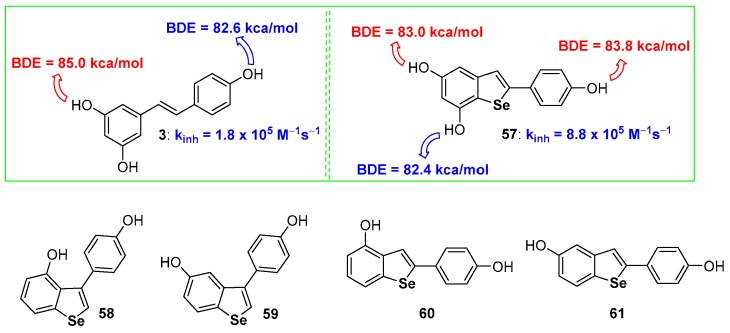
A selection of benzo[*b*]selenophenes antioxidants reported so far.

In the sulfur series, we have shown that the aromatization of dihydrobenzo[*b*]thiophenes to benzo[*b*]thiophenes is beneficial for the chain breaking antioxidant activity [68]. Among the few examples available of benzo[*b*]selenophenes used as antioxidants, derivatives 57–61 are probably the more interesting [95,96] (Figure 15) whose skeletons can be associated with that of natural resveratrol (compound 3 Figure 1). Compounds 58–61 [96], and other related derivatives were prepared and tested for their antiproliferative activity, while the peroxides quencher ability was tested using, mainly, qualitative approaches that have not been considered in this focussed review.

On the other hand, compound 57 showed a remarkable chain-breaking antioxidant activity better than the natural inspiring model 3 [95]. After the insertion of the selenium atom the OH involved in the H• transfer process, i.e., that with the lower BDE, was the OH on C7, *ortho* to the selenium atom, while the more reactive in resveratrol 3 is the OH on C4′, activated by the *para*-conjugated double bond. Despite being measured with two different methodologies, dihydroselenophenes, like 52 or 53 [94], react with ROO• radicals faster than selenophene 57 [95]. This seems to be in contrast with the result achieved with sulfur analogues that, after aromatization, increased their antioxidant activity. In our opinion this incongruity in only apparent since two different oxidation mechanisms are operative. In dihydroselenophenes 52 and 53 the mechanism described in Figure 12 blue frame is working, with the initial oxidation at selenium and ‘in the solvent cage’ extraction of H• as the final step. On the other hand, benzoselenophene 57 reasonably reacts with peroxyl radicals ROO• by a direct H• extraction from ArOH because the oxidation at selenium and formation of a R_2_Se=O derivative is not more feasible after aromatization. In other words, very probably, also for hydroxy benzo[*b*]selenophenes aromatization increases the stabilization of the corresponding ArO•, mainly by σ-hole issues, but the oxidation at Selenium in dihydrobenzo[*b*]selenophenes remains a favourite path.

### 2.3. Chalcogens in Tocopherol Skeletons

**Figure 16:**
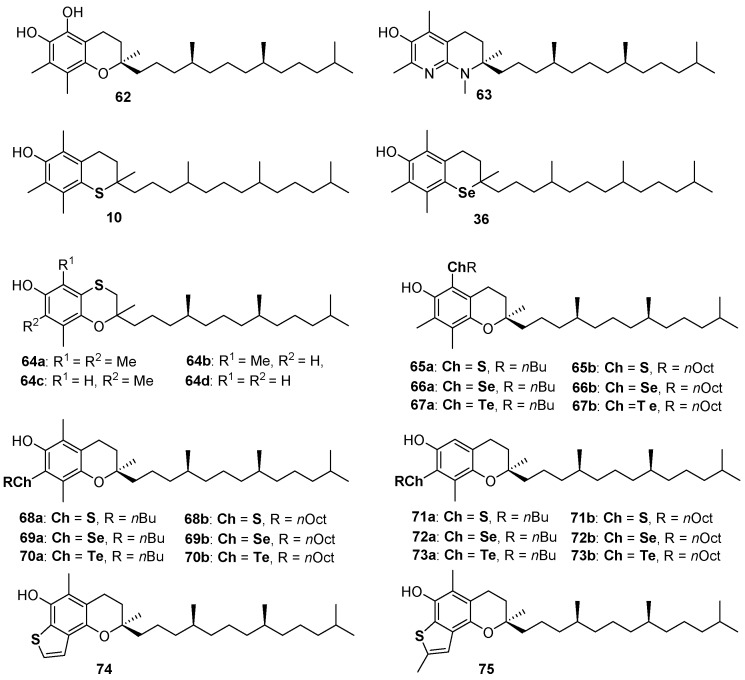
A selection of tocopherols analogues prepared so far.

α-Tocopherol (α-TOH, 1a Figure 1) is the main component of Vitamin E and the more potent lipophilic phenolic antioxidant known in nature [6,7,8,9,10,11]. Despite the role of tocopherols at the biological level still being controversial [97,98,99], their antioxidant activity is clear as well as the ROO• quenching mechanism operated by these compounds. Thus, researchers involved in the design, synthesis and application of new bio-inspired synthetic antioxidants use α-TOH as a model to imitate [100], a target to reach or, possibly, to overcome [29,34]. For example, derivative 62 was prepared to add the features of a catechol residue to that of 1a [29], while compound 63, exploiting the great ED effect of the *para*-amino substituent and the pyridinol ring ability to avoid the spontaneous reaction molecular oxygen, likely showed better antioxidant performances among α-TOH analogue with *k_inh_* ≈ 6 × 10^7^ M^−1^s^−1^, up to 20 times higher than the natural model [34] (Figure 16). We already discussed the properties of thiotocopherol 10 [45], and selenotocopherol 36 [77] and the reasons for the decreased activity observed for these analogues. Having demonstrated the possibility to obtain benzoxathiines antioxidants, we adapt the synthetic procedure for the preparation of (2-*ambo*-4′*R*,8′*R*)-tocopherol derivatives 64a–d possessing the aromatic skeletons of α-, β-, γ- and δ-tocopherol, i.e., the four saturated components of Vitamin E [101]. As previously explained, due to the flexibility of the benzoxathiine ring, compounds 64a–d were less potent than corresponding analogues 1a–d. Additionally, for these compounds also, BDE decreases, and *k_inh_* increases, with the number of methyl groups being 64a (analogue to α-TOH) the more potent and 64d (analogue to δ-TOH) the less potent chain breaking antioxidant in this series [101] (Figure 16).

Derivatives 10, 36 and 64 were not assembled using a pre-organized chromane skeleton and, indeed, required significant synthetic efforts. On the other hand, derivatives 65–73 were prepared to introduce sulfur, selenium or tellurium alkyl residues in the skeleton of commercially available β-, γ- and δ-tocopherol exploiting for functionalizations the unsubstituted position(s) on the aromatic ring [102,103]. Tellurium compounds like 67, 70 and 73 showed inhibition rates *R_inh_* for the reaction with ROO•, similar to those of α-TOH, but, differently from α-TOH, can be regenerated by a co-reductant thiol with extra-long inhibition times *T_inh_*. After learning that a benzo[*b*]thiophene is probably the best sulfur-containing residue to be inserted *ortho* to a phenolic OH involved in the H• transfer process, we synthesized derivatives 74 and 75 as the result of five consecutive electrophilic processes occurring one-pot from carefully designed benzoxathiines [104]. Indeed, benzo[*b*]thiophenes 74 and 75 showed *k_inh_* of 6 × 10^6^ M^−1^s^−1^ and 9 × 10^6^ M^−1^s^−1^ respectively, to the best of our knowledge, the better performances ever reported for a sulfur containing phenolic antioxidant. Additionally, as already reported for derivative 63 [34], we demonstrated that the structural modification brought about the construction of the thiophene ring can be well tolerated at biological level and compounds 74 and 75 showed a binding with α-tocopherol transfer protein (α-TTP) very similar to that of the natural substrate α-TOH [104].

**Figure 17:**
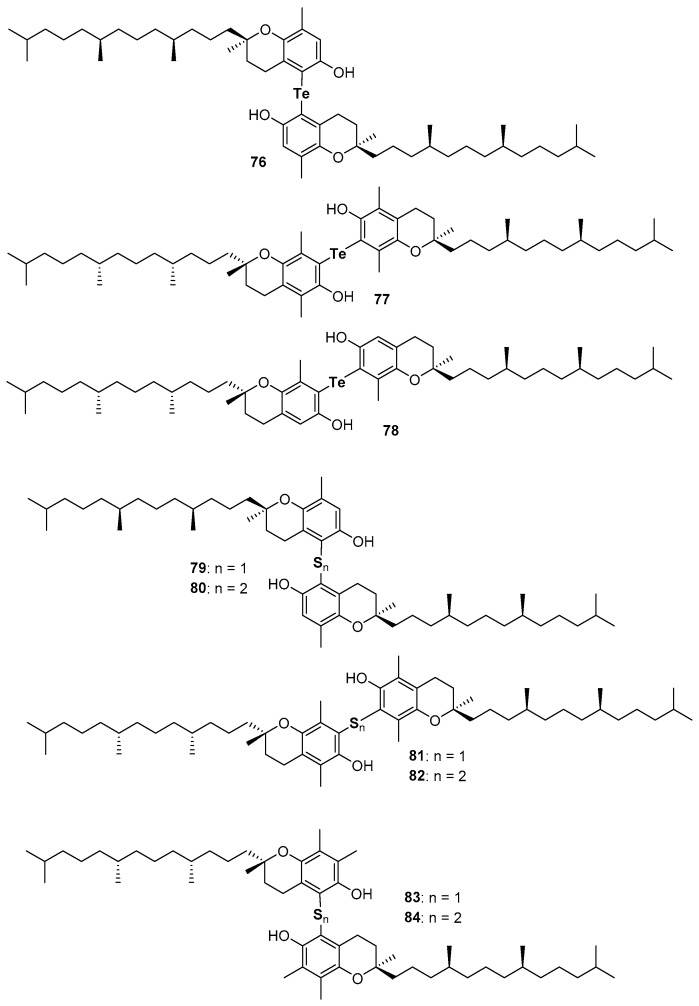
Chalcogens containing ditocopherols prepared so far.

**Figure 18:**
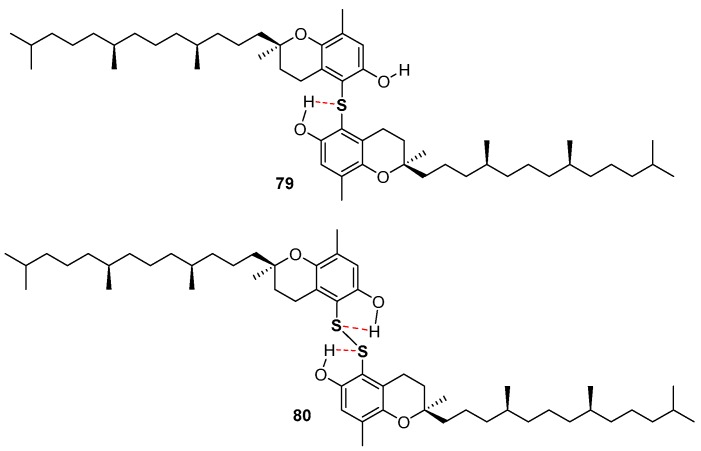
Role of intra-HB in δ,δ-ditocopheryl sulfide 79 and δ,δ-ditocopheryl disulfide 80.

Very recently, the ability in the manipulation of heavy chalcogen reagents allowed the preparation of di-tocopheryl tellurides 76–78 [105] and ditocopheryl sulfides 79, 81, 83 and ditocopheryl disulfides 80, 82, 84 [106] (Figure 17). Ditocopheryl tellurides 76–78 showed, as expected, a very good inhibition rate *R_inh_* and catalytic activity inhibition times *T_inh_* as already reported in similar Te-substituted phenols. Ditocopheryl sulfides and disulfides were generally less reactive towards ROO• than the corresponding tocopherols. This was expected in light of previously discussed considerations about *ortho*-OH effect of acyclic sulfur substituents [43] (see Figure 5 and Figure 7). In more detail, for each couple (δ,δ-, γ,γ-, β,β-) disulfides are less active than sulfides and δ,δ-ditocopheryl sulfide 79 was the compound with the higher *k_inh_*. The better activity of sulfides than disulfides can be rationalised, as confirmed by FT-IR studies and supported by *ab-initio* calculations, considering that in disulfides both phenolic OH are engaged in an intra-HB, while in sulfides only one phenolic OH is engaged, while the other can efficiently participate to the H• transfer process. Rationalization of the superiority of 79 when compared with 81 and 83, despite the lack of one ED methyl group is less trivial. However, molecular dynamic calculations showed that in the absence of a methyl group *ortho* to the phenolic OH, as it occurs in δ,δ-79, the intra-HB strength is further decreased (Figure 18). In other words, the additional *ortho*-methyl group in sulfides γ,γ-81 or β,β-83 favours conformations with the phenolic OH protons pointing toward the sulfur atom, increasing the intra-HB strength hence reducing the chain breaking antioxidant activity [106].

## 3. Conclusions

The fine-tuning of the chalcogens stereoelectronic features, the ability in design and synthesis of chalcogens containing compounds and the skills to meticulously evaluate the chain-breaking antioxidant activity allowed the preparation of a plethora of new polyphenolic antioxidants. The actual role of heavy chalcogens (S, Se and Te) in modulating the chain-breaking antioxidant activity has been reviewed and discussed. Very few insights are available about the toxicity/biocompatibility of the great part of the derivatives shown here. Since many of these compounds derive from natural precursors, the study of eventual change of toxicity after the introduction of the heavy chalcogens (above all Se and Te) would be of great interest. Additionally, the medicine industry always searches for new antioxidants with better chemical or biological properties, thus we hope this review could stimulate the readers interest and curiosity to face these research challenges.
